# Antibacterial activity of cinnamon essential oil and its main component of cinnamaldehyde and the underlying mechanism

**DOI:** 10.3389/fphar.2024.1378434

**Published:** 2024-03-11

**Authors:** Chengjie Shu, Ling Ge, Zhuohang Li, Bin Chen, Shengliang Liao, Lu Lu, Qinlin Wu, Xinyi Jiang, Yuhan An, Zongde Wang, Man Qu

**Affiliations:** ^1^ School of Forestry, Jiangxi Agricultural University, Nanchang, China; ^2^ Natural Daily Chemical Research Laboratory, Nanjing Institute for Comprehensive Utilization of Wild Plants, Nanjing, China; ^3^ School of Public Health, Yangzhou University, Yangzhou, China

**Keywords:** *in vivo* and *in vitro*, antibacterial mechanisms, CIEO and CID, P38 signaling, antimicrobial peptides

## Abstract

**Background:** Plant essential oils have long been regarded as repositories of antimicrobial agents. In recent years, they have emerged as potential alternatives or supplements to antimicrobial drugs. Although literature reviews and previous studies have indicated that cinnamon essential oil (CIEO) and its major component, cinnamaldehyde (CID), possess potent antibacterial activities, their antibacterial mechanisms, especially the *in vivo* antibacterial mechanisms, remain elusive.

**Methods:** In this study, we utilized the *in vivo* assessment system of *Caenorhabditis elegans* (*C. elegans*) to investigate the effects and mechanisms of high dose (100 mg/L) and low dose (10 mg/L) CIEO and CID in inhibiting *Pseudomonas aeruginosa* (*P. aeruginosa*). In addition, we also examined the *in vitro* antibacterial abilities of CIEO and CID against other common pathogens including *P. aeruginosa* and 4 other strains.

**Results:** Our research revealed that both high (100 mg/L) and low doses (10 mg/L) of CIEO and CID treatment significantly alleviated the reduction in locomotion behavior, lifespan, and accumulation of *P. aeruginosa* in *C. elegans* infected with the bacteria. During *P. aeruginosa* infection, the transcriptional expression of antimicrobial peptide-related genes (*lys-1* and *lys-8*) in *C. elegans* was upregulated with low-dose CIEO and CID treatment, while this trend was suppressed at high doses. Further investigation suggested that the PMK-1 mediated p38 signaling pathway may be involved in the regulation of CIEO and CID during nematode defense against *P. aeruginosa* infection. Furthermore, *in vitro* experimental results also revealed that CIEO and CID exhibit good antibacterial effects, which may be associated with their antioxidant properties.

**Conclusion:** Our results indicated that low-dose CIEO and CID treatment could activate the p38 signaling pathway in *C. elegans*, thereby regulating antimicrobial peptides, and achieving antimicrobial effects. Meanwhile, high doses of CIEO and CID might directly participate in the internal antimicrobial processes of *C. elegans*. Our study provides research basis for the antibacterial properties of CIEO and CID both *in vivo* and *in vitro*.

## 1 Introduction

Cinnamon occupies a significant position as one of the most crucial spices and traditional herbal medicines worldwide (Gruenwald et al., 2010). Derived from cinnamon, cinnamon essential oil (CIEO) comprises volatile constituents, notably cinnamaldehyde (CID), alongside non-volatile elements such as polysaccharides, polyphenols, flavonoids, and various compounds (Lu et al., 2020). Investigations affirm that the polyphenols inherent in CIEO can potentiate insulin sensitivity, thereby aiding in the management of glucose intolerance (Chen et al., 2012; Lu et al., 2020). Furthermore, the flavonoid compounds within CIEO serve a dual role by preserving food and bestowing antioxidant properties, endowing therapeutic benefits for metabolic disorders by mitigating damage inflicted by free radicals (Gruenwald et al., 2010; Lu et al., 2020; Abeysekera et al., 2022). CID, a prominent constituent of CIEO, exhibits antiplatelet activity, impeding thrombus contraction (Tognolini et al., 2006). Additionally, an additional study posits potential benefits of CID in anti-hyperglycemic and lipid-lowering applications (Subash Babu et al., 2007). Research by Ka et al., 2003 has unveiled the cytotoxic effects of cinnamon aldehyde on HL-60 cancer cells, thus inhibiting their proliferation. More significantly, according to reports, cinnamon oil has been documented to be safe for treating human oral candidiasis infections and gastritis caused by *Helicobacter pylori* (Sim et al., 2019). Notwithstanding several studies highlighting the antibacterial properties of CIEO and its principal component, CID, the precise antibacterial mechanisms of both substances, both *in vitro* and *in vivo*, remain unclear.

*Pseudomonas aeruginosa* (*P. aeruginosa*), a Gram-negative rod-shaped bacterium affiliated with the *Pseudomonadaceae* family, is distinguished for its pathogenicity, especially in individuals with compromised immune function, thus establishing it as a notable opportunistic human pathogen (Stover et al., 2000; Sathe et al., 2023). It has been reported that *P. aeruginosa* may cause various serious infections, including sepsis, endocarditis, pneumonia, urinary tract infections, peritonitis, and other health hazards (Reyes et al., 2023). In studies focused on the innate immunity of *C. elegans*, *P. aeruginosa* is commonly used as a representative pathogen to investigate host-pathogen interactions, infection, and anti-infection mechanisms (Yu et al., 2018; Martineau et al., 2021).

*Caenorhabditis elegans* (*C. elegans*), characterized by its small size, short lifecycle, transparent body, suitability for large-scale genetic research and phenotype analysis, and sensitivity to various environmental exposures, has proven to be a robust invertebrate model for investigating host-pathogen interactions (Martineau et al., 2021; Qu et al., 2023a; Ma et al., 2023). Like other invertebrates, *C. elegans* relies entirely on its innate immune system to defend against pathogens. The innate immune system of *C. elegans* employs evolutionarily conserved signaling pathways, making it a widely used model for research on infections caused by various fungi and bacterial pathogens, including *P. aeruginosa* (Yu et al., 2018; Du et al., 2020). In *C. elegans*, activated innate immune responses result in the secretion of antimicrobial proteins to kill pathogens (Aballay and Ausubel, 2002; Martineau et al., 2021).

In this study, we utilized the model organism *C. elegans* to investigate the *in vivo* antibacterial effects and potential mechanisms of CIEO and its main component CID. Additionally, we validated the antibacterial effects of CIEO and CID on common bacteria, including *P. aeruginosa*, through *in vitro* experiments. Our research demonstrated that low doses of CIEO and CID treatment can inhibit *P. aeruginosa* infection in *C. elegans* by promoting the expression of certain antimicrobial peptides (LYS-1 and LYS-8). Contrary to expectations, high concentrations of CIEO and CID treatment did not lead to increased expression of more antimicrobial peptides. We speculate that high concentrations of CIEO and CID may directly inhibit the accumulation of *P. aeruginosa* within the nematode. Further investigation suggested that the PMK-1 mediated p38 signaling pathway may be involved in the regulation of CIEO and CID during nematode defense against *P. aeruginosa* infection. Moreover, *in vitro* experiments confirmed the effective antibacterial properties of CIEO and CID against various pathogens. Our data suggested the potential of CIEO and CID in inhibiting *P. aeruginosa* infection in the host.

## 2 Materials and methods

### 2.1 Separation and identification of CID

The CIEO used in this study was purchased from Longqing Agricultural Technology Development Co., Ltd. CID is obtained by separating it from CIEO through vacuum distillation using a vacuum distillation apparatus (RFJL-02, Ruitaifeng Technology Co., Ltd, China). The specific steps are as follows: under the conditions of a pressure of 23 kPa and a reflux ratio of 2:1, vacuum distillation is performed on CIEO. After a full reflux for 1 h, fractions are collected at different temperature ranges of 85°C–95°C, 95°C–105°C, 105°C–115°C, 115°C–120°C, 120°C–125°C, and 125°C–130°C from the top of the column. The CID content in each fraction is analyzed using a gas chromatography and mass spectrometry (7000E, GC-MS, Agilent Technologies, Inc United States). The chromatographic column used is DB-5MS. The initial temperature is set at 80°C and held for 5 min, followed by a temperature ramp of 5°C/min to reach 200°C, with a subsequent hold for 5 min. Helium is employed as the carrier gas, with the injection port temperature at 250°C and the FID detector temperature at 280°C. The injection volume is 1 μL. The EI ion source is maintained at 230°C, the quadrupole temperature at 150°C. The electron energy is set at 70 eV, and the entire spectrum is scanned.

### 2.2 Nematode maintenance

The wild-type N2 strain was used in this study, cultivated on nematode growth medium (NGM) plates nourished with *E. coli* (*E. coli*) OP50 as the food source (Yu et al., 2018). To procure an ample cohort of young worms for bacterial infection, we exposed the gravid hermaphroditic nematodes to a lytic solution comprising 0.45 M NaOH and 2% HClO to liberate ova from their corporeal confines (Qu et al., 2023b). Following this, the ova were collected and translocated onto the substrate of a pristine NGM plate, fostering their maturation into synchronized youthful nematodes.

### 2.3 Preparation and infection of *P. aeruginosa*

The *P. aeruginosa* strains utilized encompassed PA14 and PA14:GFP. PA14 stands as a frequently employed *P. aeruginosa* variant for the exploration of innate immunity dynamics in *C. elegans* (Yu et al., 2018). The strains underwent cultivation in Luria broth. The complete surface of the lethality-inducing plates for *P. aeruginosa* PA14 or PA14:GFP was prepared by inoculating them onto adapted NGM (consisting of peptone 3.5 g, sodium chloride 3.0 g, calcium chloride 0.111 g, magnesium sulfate 0.12 g, cholesterol 0.005 g, potassium phosphate monobasic 3.4 g, agar 17.0 g per 1 L) lethality-inducing plates. Post-seeding, the lethality-inducing plates underwent incubation at 37°C for a duration of 24 h, followed by an additional 24 h of incubation at 25°C. Young nematodes were translocated onto lethality-inducing plates to commence *P. aeruginosa* PA14 infection, maintained at 25°C for a duration of 24 h (Wang et al., 2023). Three independent replicates were executed.

### 2.4 Locomotion behaviors and lifespan assays

Head thrashes, body bends, and lifespan were commonly used as endpoints to evaluate the pharmacological effects of certain drugs on *P. aeruginosa* PA14 infection in *C. elegans* (Chelliah et al., 2018; Yu et al., 2018). Following a 24-h *P. aeruginosa* PA14 infection, locomotion behavior was assessed. Each examined nematode was transferred to an agar plate, with 50 μL of K medium. Following a 1-min recovery period, body bends were employed to evaluate locomotion behavior. A head thrash is a behavior observed in *C. elegans*, where the worm rapidly alters the bending direction at the mid-region of its body (Qu et al., 2023c). The calculation of a body bend involves measuring the wavelength of the nematode’s movement (Qu et al., 2024). Lifespan was defined as the duration of survival for animals from the egg stage (day 0) to death on the final day (Qu et al., 2022). The lifespan assay was conducted at 20°C. Animals were transferred daily during the initial 7 days. Three independent replicates were executed.

### 2.5 Analysis of worm bacterial colony-forming unit

Nematode bacterial colony-forming unit analysis followed the following procedure (Yu et al., 2018): Following a 24-h infection of nematodes with *P. aeruginosa* PA14, 6 replicates of 10 nematodes each were moved to an M9 solution containing 25 mM levamisole to induce nematode paralysis and halt pharyngeal pumping. The nematodes were then transferred to an NGM plate containing ampicillin (1 mg/mL) and gentamicin (1 mg/mL) for 15 min to remove *P. aeruginosa* adhered to the animal body. The nematodes were lysed using a motorized pestle, and the lysates were sequentially diluted in M9 solution and plated on Luria Bertani plates containing rifampicin (100 μg/mL) to isolate *P. aeruginosa* PA14. Following an overnight incubation at 37°C, the colonies were enumerated to calculate the CFU per nematode. Three independent replicates were executed.

To verify the accumulation of *P. aeruginosa* in the intestinal lumen, we employed the *P. aeruginosa* PA14:GFP to directly observe the accumulation in nematodes. The data were presented as the relative fluorescence intensity of *P. aeruginosa* PA14:GFP in intestinal lumens, normalized to the intestinal autofluorescence of nematodes. Fifty worms were examined in each group, and the experiment was replicated three times.

### 2.6 Pharmacological assays

*In vivo* assay: Following *P. aeruginosa* PA14 infection, the nematodes were exposed to CIEO or CID for posttreatment for 24 h. CIEO or CID treatment concentrations for *in vivo* tests included 10 and 100 mg/L. The selection of concentrations was primarily guided by a previously published report (Lu et al., 2020). Three replicates were conducted.

*In vitro* assay: The used bacterial strains of *Candida albicans* (*C*. *albicans*) ATCC 10231, *E*. *coli* ATCC 25922, *S. aureus* (*S*. *aureus*) ATCC29213, *P*. *aeruginosa* ATCC 27853, and *B. subtilis* (*B. subtilis*) ATCC 6633 were obtained from Shanghai Beinuo Biotechnology Co., Ltd., China. CIEO or CID treatment concentrations for *in vitro* tests included 2.5–40 mg/mL. The antibacterial zone test followed the following procedure: A 800 μL bacterial suspension (2 × 10^8^ CFU/mL) was evenly spread on the surface of a 90 mm culture medium (Corning, United States), and a 6 mm antibacterial disc was placed on the corresponding medium. A 10 μL sample was applied to the antibacterial disc, and left to stand for 10 min. Incubation was carried out at 37°C for 24 h, with three replicates for each group.

Minimum inhibitory concentration (MIC) is defined as the lowest concentration of an antimicrobial that will inhibit the visible growth of a microorganism after overnight incubation (Andrews, 2001). Minimum bactericidal concentration (MBC) was determined as the lowest concentration at which 99.9% of the bacterial inoculum was eradicated, visually assessed by the absence of growth on the agar plate where the aliquots were placed (Fernandes et al., 1985). CIEO and CID at concentrations of 2.5–40 mg/mL were prepared in dimethyl sulfoxide (DMSO). The MIC and MBC followed the following procedure: 1 mL of liquid culture was mixed with 500 μL of different concentrations of CIEO or CID, along with 500 μL of bacterial suspension (2 × 10^6^ CFU/mL). The test tubes were incubated at 37°C on a shaker at 180 rpm for 24 h, and 50 μL of the clarified tube liquid were evenly spread on the corresponding solid culture medium, and incubated at 37°C for 24 h. The absence of visible bacterial growth indicates MBC. Each experiment was repeated three times.

### 2.7 Detection of superoxide dismutase and catalase activity

SOD and catalase CAT are considered the first line of defense for cells against oxidative damage, and their activity levels can reflect the extent to which cells are influenced by external stressors (Qu et al., 2018). SOD catalyzes the conversion of superoxide anions into H_2_O_2_ and O_2_, serving as the primary substance for eliminating free radicals within organisms. CAT, on the other hand, is an enzyme responsible for eliminating H_2_O_2_. Through synergistic action, both SOD and CAT work together to maintain a balanced level of free radicals within cells, preventing cellular toxicity caused by free radicals. After exposure, nematodes were thoroughly washed with M9 buffer and then added to 9 mL of ice-cold phosphate buffer (pH = 7.2). A 10% tissue homogenate is prepared, followed by centrifugation at 3,000 rpm for 15 min in a low-temperature, high-speed centrifuge. The supernatant is collected and stored at −20°C. SOD activity is determined using the xanthine oxidase method, and CAT activity is measured using the ammonium molybdate method. The SOD (Catalog Number: A001-3-2) and CAT (Catalog Number: A007-1-1) assay kits used in this study were obtained from Nanjing Jiancheng Bioengineering Institute, and the procedures are carried out according to the instructions provided with the kits.

### 2.8 Determination of 2,2′-azino-bis (3-ethylbenzothiazoline-6-sulfonic acid) (ABTS) radical scavenging ability

The ABTS assay is widely used for the determination of the antioxidant capacity of both hydrophilic and lipophilic substances. ABTS solution (7 mmol/L) was mixed in a 1:1 ratio (by mole) with potassium persulfate solution (2.45 mmol/L), and the mixture was allowed to react fully in the dark for 12–16 h. The absorbance was maintained at 0.709 ± 0.02 (734 nm). 2 mL of various concentrations (0.5, 1, 2, 4, 8, and 16 mg/mL) of CIEO or CID were thoroughly mixed with 2 mL of ABTS^+^ solution, and left to stand for 10 min. The absorbance of the samples was measured at a wavelength of 734 nm, denoted as A_1_, with the control group (without ethanol) represented as A_0_. Butylated hydroxyanisole (BHA) was used as the positive control. The experiment was repeated three times, and the average was calculated using the formula: ABTS Free Radical Scavenging Rate (%) = (A_0_ - A_1_)/A0 × 100%.

### 2.9 Ferric ion reducing antioxidant power analysis

The principle of the FRAP method for determining total antioxidant capacity is based on the ability of antioxidants under acidic conditions to reduce iron-tripyridyltriazine (Fe-TPTZ), producing a blue-purple color. Subsequently, the absorbance is measured at 593 nm, serving as an indicator of the total antioxidant capacity in the sample (Floridi et al., 2009). Preparation of TPTZ Working Solution: TPTZ solution (10 mmol/L) was mixed with FeCl_3_ solution (20 mmol/L) and acetate buffer solution (0.3 mol/L) in a 1:1:10 (volume ratio), prepared and used immediately.

FeSO_4_ Standard Curve: FeSO_4_ solution was mixed with the TPTZ working solution at a ratio of 1:3, thoroughly mixed, and allowed to react at 37°C for 30 min. The absorbance is measured at 593 nm to construct a standard curve. The reduction potential experiment is conducted with reference to previous studies. The corresponding FRAP values are obtained through the FeSO_4_ standard curve.

### 2.10 Real-time quantitative reverse transcription PCR (qRT-PCR) analysis

qRT-PCR procedures were executed in accordance with previously delineated protocols (Qu et al., 2019). Following exposure, nematodes underwent thorough washing, a minimum of three repetitions, with K buffer before being transferred to a 1.5 mL RNA enzyme-free EP tube for total RNA extraction, utilizing Trizol (Sigma-Aldrich, St. Louis, MO, United States). The total RNAs obtained were purified through the RNeasy purification kit (Invitrogen, United States). Subsequently, the purified RNAs underwent reverse transcription into complementary DNA (cDNA) utilizing the PrimeScript™ RT reagent kit (Takara, Japan). This was followed by the execution of qRT-PCR to amplify PCR products using the SYBR Green qRT-PCR master mix (Toyobo, Japan) with the StepOnePlus™ real-time PCR system (Applied Biosystems, United States). Within this investigation, *tba-1*, encoding α-tubulin, was selected as the reference gene, and the relative expression of target genes compared to *tba-1* was subsequently computed. Three concurrent reactions were arranged for each experimental group. The precise primer sequences for qRT-PCR can be found in Supplementary Table S1.

### 2.11 RNA interference

Inducing RNA interference (RNAi) in nematodes was achieved by feeding them with bacteria engineered to express double-stranded RNA (dsRNA), a widely adopted method (Kamath and Ahringer, 2003). In this study, L1 stage worms were placed on NGM agar plates containing bacteria expressing dsRNA, following the procedure described previously (Qu et al., 2023b). The primer information for constructing the RNAi vector is available in Supplementary Table S2. To validate the interference effect of dsRNA in the RNAi nematodes, we assessed the effectiveness of RNAi through qRT-PCR, measuring the mRNA expression levels of the targeted gene (data not shown).

### 2.12 Statistical analysis

Statistical analysis was performed using SPSS 25.0 (SPSS Inc., Chicago, United States). The parameters utilized in this study were defined as continuous variables. The homogeneity of variance was evaluated using the *Agostino D* test. Subsequently, to examine differences among groups, a one-way analysis of variance (ANOVA) was executed, followed by the SNK-q test for multiple comparisons. A confidence level of 0.05 was considered statistically significant.

## 3 Results

### 3.1 Characteristics of CIEO and CID used in this study

**FIGURE 1:**
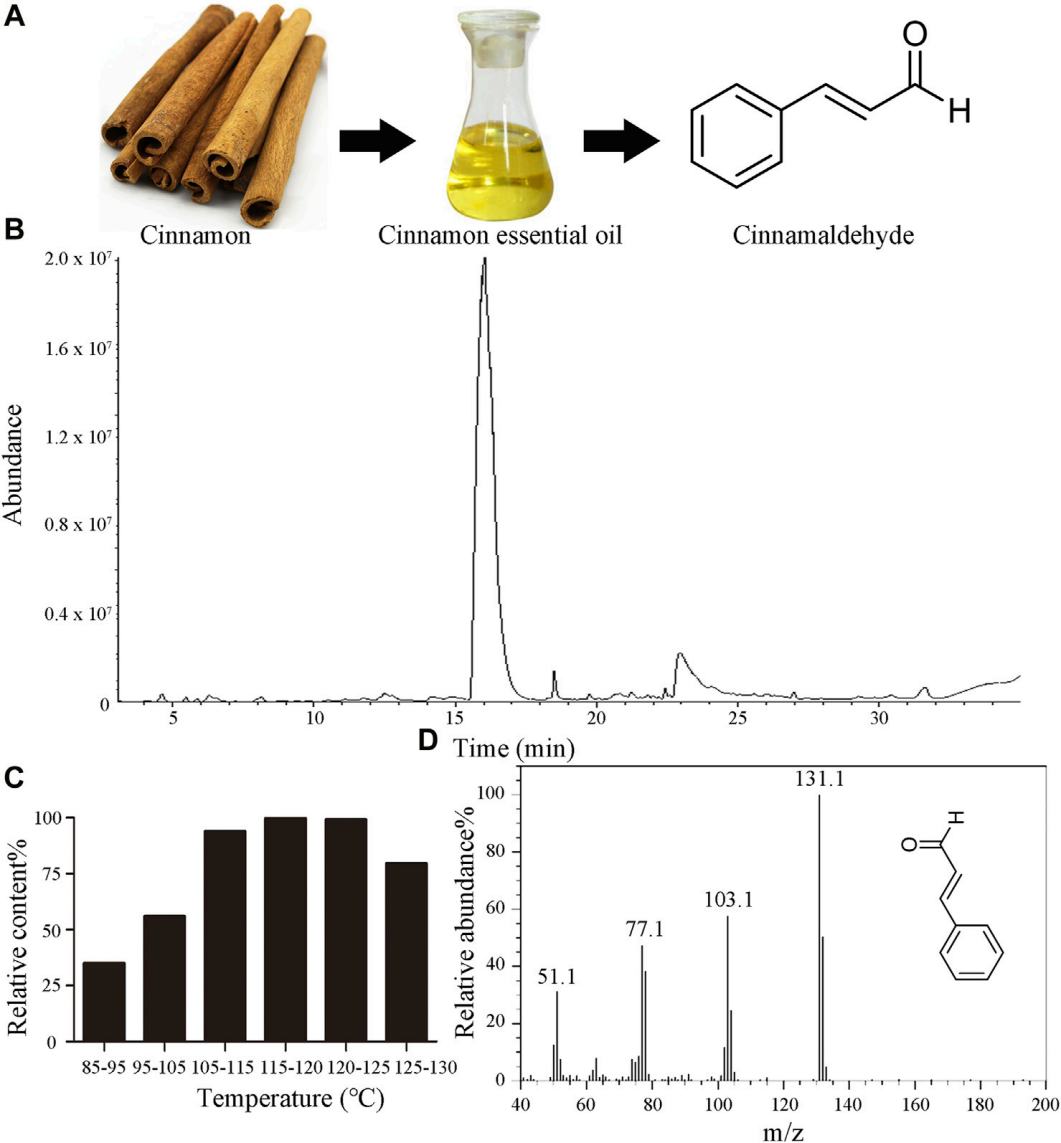
Characteristics of CIEO and CID **(A)**. Chemical structure of CID **(B)**. GC-MS total ion chromatogram of CIEO **(C)**. CID content in each fraction of the vacuum distillation of CIEO **(D)**. Mass spectrum of CID.

The chemical structure of CID is depicted in Figure 1A. GC-MS analysis indicated the chemical composition of CIEO and the content of CID in each fraction. The total ion chromatogram of CIEO is shown in Figure 1B, and the chemical composition is presented in Supplementary Table S3, while the CID content in each fraction is detailed in Figure 1C. As observed in Supplementary Table S3, a total of 15 components were detected, with the major constituents being CID (86.07%), 2-methoxycinnamaldehyde (6.25%), α-cubebene (1.04%), diisobutyl phthalate (1.02%), and coumarin (0.67%). According to Figure 1C, it could be observed that within the temperature range of 85°C–120°C, the CID content in each fraction increases with the rise in temperature. However, in the range of 115°C–130°C, there was a decreasing trend in CID content with increasing temperature, possibly due to the outflow of reconstituted components such as methoxy cinnamaldehyde. The CID content in the 115°C–120°C fraction was 99.78%, and in the 120°C–125°C fraction, it was 99.11%. Hence, it can be inferred that the CID content in the 115°C–125°C fraction exceeds 99.00%. Figure 1D displays the mass spectrum of CID obtained after vacuum distillation.

### 3.2 CIEO or CID treatment attenuated the decline in locomotion ability and the reduction in lifespan induced by *P. aeruginosa* PA14-infected nematodes

**FIGURE 2:**
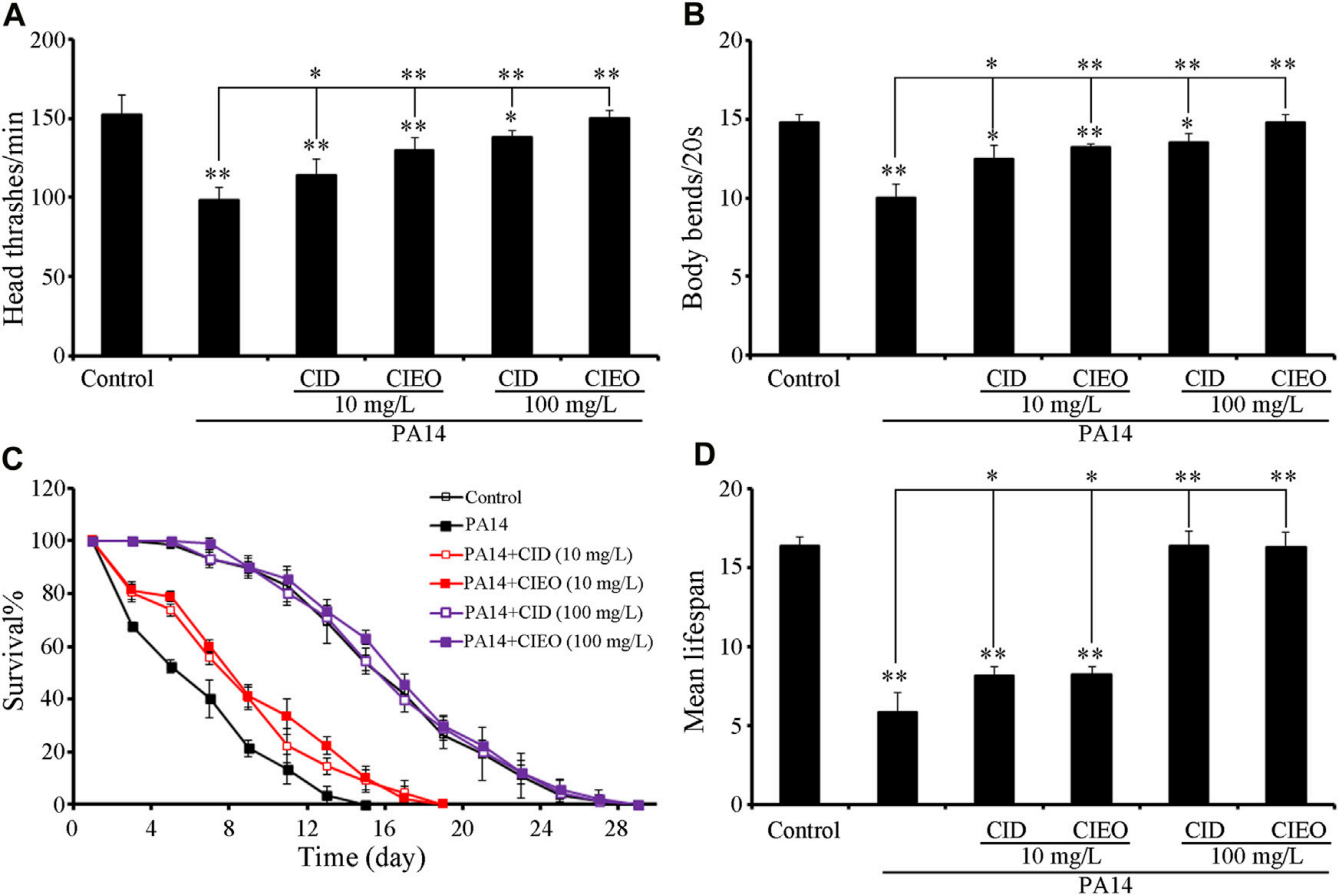
Effects of CIEO or CID treatment on the locomotion behavior and lifespan of *P. aeruginosa* PA14-infected nematodes. Effects of CIEO or CID treatment in *P. aeruginosa* PA14-infected nematodes on **(A)**. Head thrashes, **(B)**. Body bends, **(C)**. Survival %, and **(D)**. Mean lifespan. Exposure to *P. aeruginosa* commenced at the L4 stage and spanned a period of 24 h. Bars represent means ± SD. *p < 0.05 vs. control and **p < 0.01 vs. control.

Employing locomotion behavior and lifespan as endpoints, we explored the potential beneficial effects of CIEO and CID treatment on *P. aeruginosa* PA14 infection in *C. elegans*. The exposure of wild-type N2 to *P. aeruginosa* PA14 significantly impeded the locomotion behavior of nematodes, marked by a decrease in head thrashes and body bends (Figures 2A,B). Furthermore, exposure to *P. aeruginosa* PA14 led to a more pronounced decrease in the lifespan of wild-type nematodes (Figures 2C,D). Following posttreatment, ≥10 mg/L CID or CIEO notably enhanced the locomotion behavior and lifespan of nematodes post *P. aeruginosa* PA14 infection (Figure 2). The beneficial impact of CID or CIEO posttreatment on enhancing the locomotion behavior and lifespan of nematodes infected with *P. aeruginosa* PA14 depended on the concentration (Figure 2).

### 3.3 CIEO or CID treatment inhibited the bacterial CFU accumulation induced by *P. aeruginosa* PA14-infected nematodes

**FIGURE 3:**
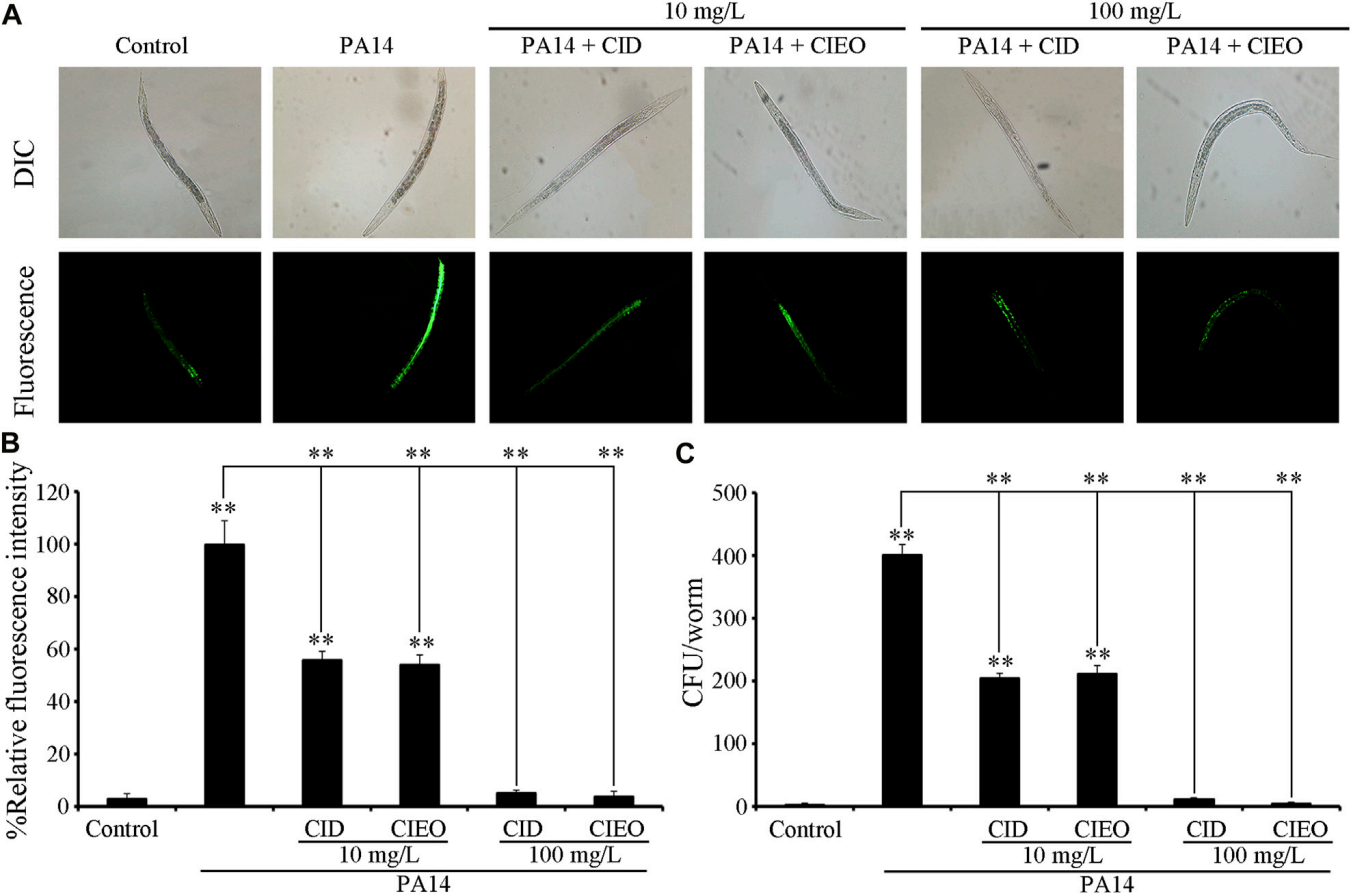
Effects of CIEO or CID treatment on the bacterial CFU of *P. aeruginosa* PA14-infected nematodes. Effects of CIEO or CID treatment in *P. aeruginosa* PA14-infected nematodes on **(A)**. Accumulation of *P. aeruginosa* PA14:GFP, **(B)**. CFU of *P. aeruginosa* PA14, and **(C)**. Fluorescence intensity of *P. aeruginosa* PA14:GFP. Exposure to *P. aeruginosa* commenced at the L4 stage and spanned a period of 24 h. Bars represent means ± SD. **p < 0.01 vs. control.

To elucidate the underlying mechanisms behind the observed advantageous effect of CIEO or CID treatment against *P. aeruginosa* PA14 infection, we initially assessed the CFU of PA14-infected nematodes. CIEO or CID treatment (≥10 mg/L) exhibited a significant impediment to the formation of elevated CFU of *P. aeruginosa* PA14 in nematodes (Figure 3B). Additionally, treatment with CIEO or CID further markedly repressed the accumulation of *P. aeruginosa* PA14:GFP in *C. elegans* (Figures 3A,C). Furthermore, the positive impact of CIEO or CID treatment on restraining the *P. aeruginosa* PA14 colony formation in nematodes was contingent upon the concentration (Figure 3).

### 3.4 Exposure to CIEO or CID altered the innate immune response homeostasis in *P. aeruginosa* PA14-infected nematodes

**FIGURE 4:**
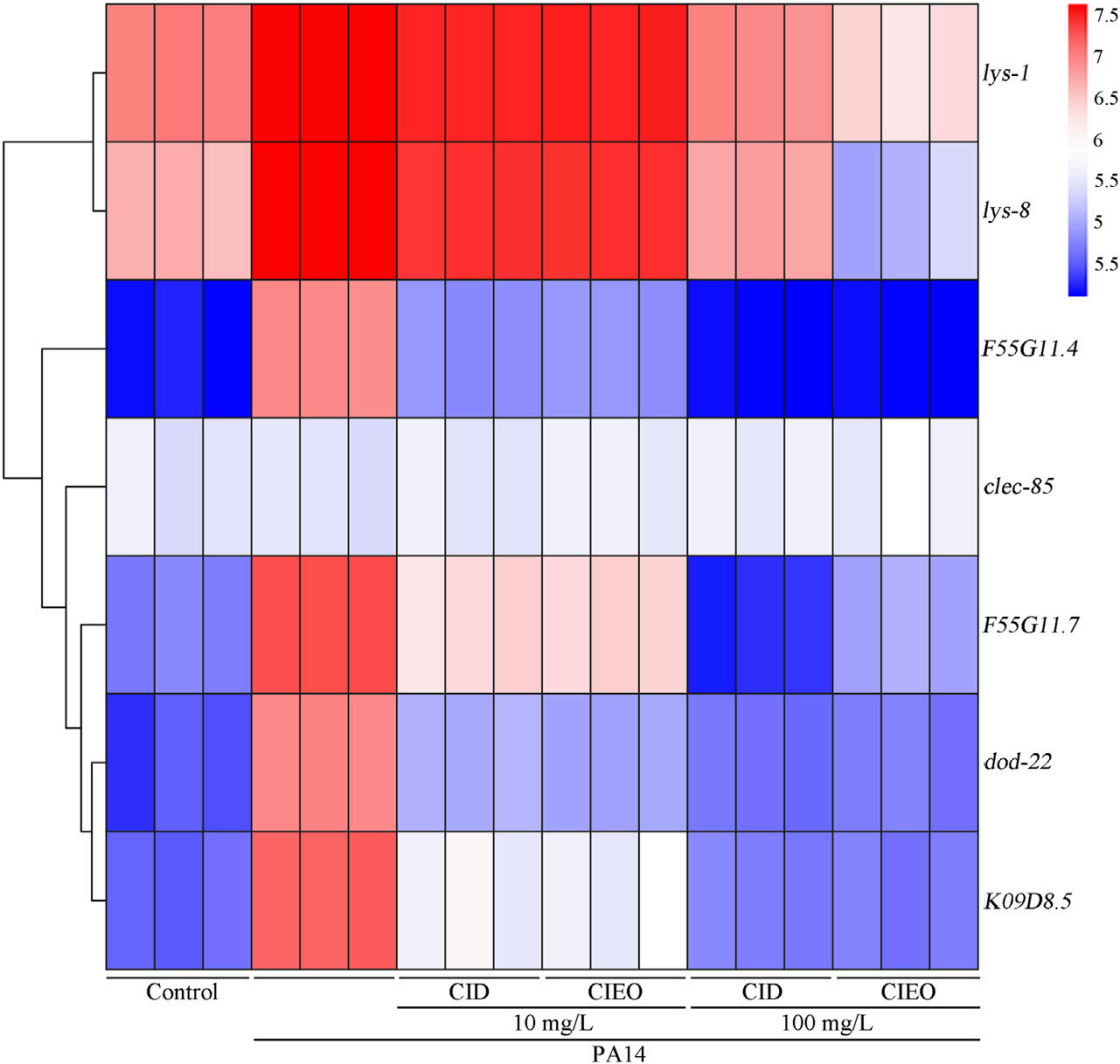
Effects of CIEO or CID treatment on the transcription levels of antimicrobial peptide related genes in *P. aeruginosa* PA14-infected nematodes. Exposure to *P. aeruginosa* commenced at the L4 stage and spanned a period of 24 h.

*lys-1* (encodes an antimicrobial effector lysozyme), *lys-8* (encodes an antimicrobial effector lysozyme), *F55G11.4* (encodes a CUB domain containing protein), *clec-85* (encodes a C-type lectin protein), *F55G11.7* (involved in innate immune response), *dod-22* (encodes a CUB domain containing protein), and *K08D8.5* (encodes a CUB domain containing protein) collectively constitute the innate immune system in *C. elegans*, orchestrating a defense mechanism against infection by *P. aeruginosa* PA14 (Mallo et al., 2002; Shapira et al., 2006; Alper et al., 2007; White and Herman, 2018; Shao and Wang, 2020; Yang et al., 2023). Our results suggested that *P. aeruginosa PA14* infection upregulated the expression levels of antimicrobial peptide-related genes, including *lys-1*, *lys-8*, *F55G11.4*, *F55G11.7*, *dod-22*, and *K09D8.5*, in *C. elegans* (Figure 4). Simultaneously, treatment with 10 mg/L CIEO or CID increased the transcription levels of *lys-1* and *lys-8* in nematodes infected with *P. aeruginosa* PA14 (Figure 4). Conversely, treatment with 10 mg/L CIEO or CID exhibited a decreasing trend in the expression levels of *F55G11.4*, *F55G11.7*, *dod-22*, and *K09D8.5* in nematodes infected with *P. aeruginosa* PA14 (Figure 4). Furthermore, this trend became more prominent with 100 mg/L CIEO or CID treatment, indicating a more significant decrease in the expression levels of *lys-1*, *lys-8*, *F55G11.4*, *F55G11.7*, *dod-22*, and *K09D8.5* compared to nematodes infected with *P. aeruginosa* PA14 (Figure 4).

### 3.5 Requirement of PMK-1 for antibacterial effect of CIEO or CID in *P. aeruginosa* PA14-infected nematodes

**FIGURE 5:**
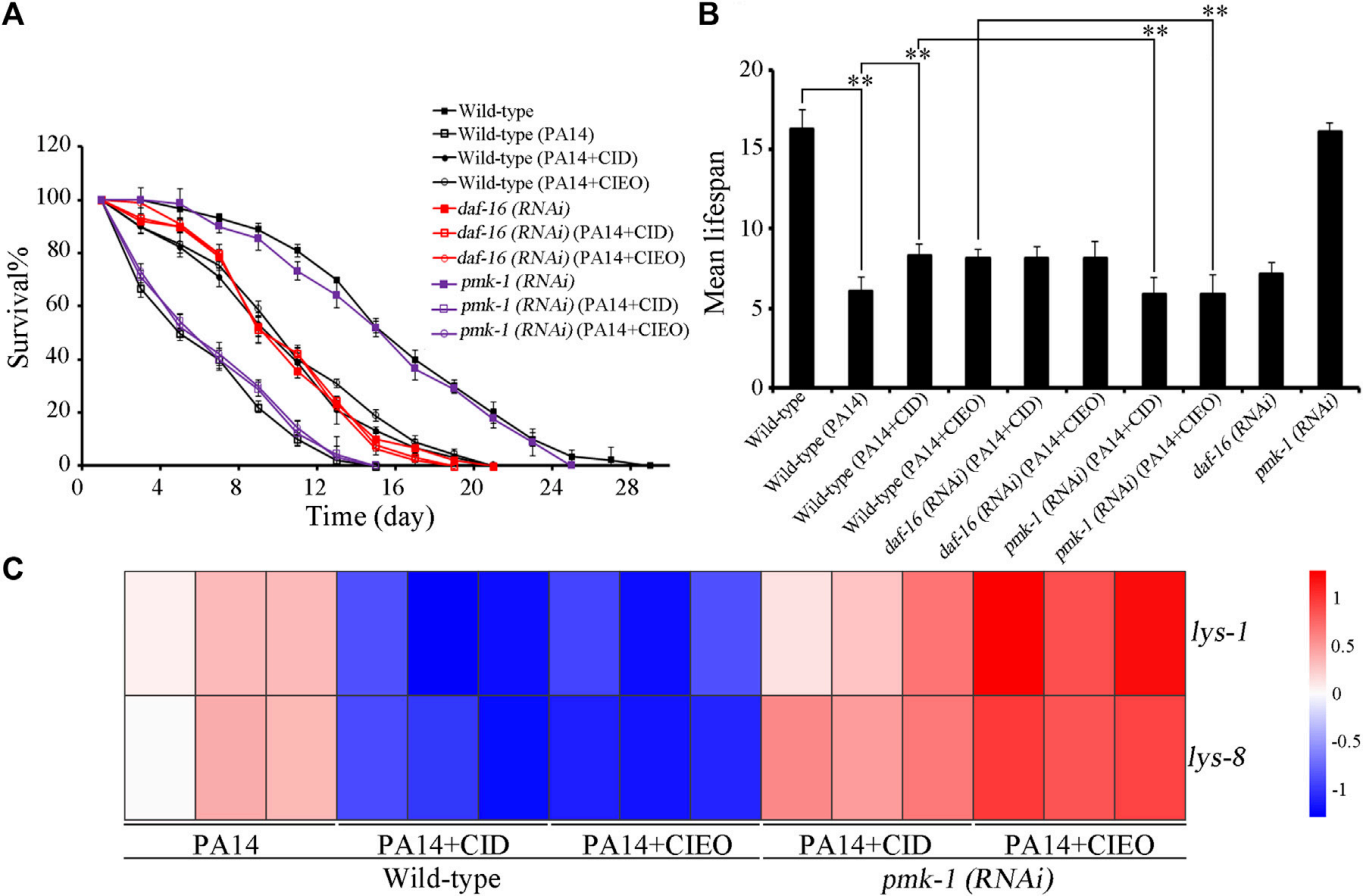
Effects of *daf-16* and *pmk-1* RNAi on beneficial role of 10 mg/L CIEO or CID treatment in *P. aeruginosa* PA14-infected nematodes. **(A)**. Effects of CIEO or CID treatment in *P. aeruginosa* PA14-infected nematodes with *daf-16* or *pmk-1* RNAi in **(A)**. Survival %, and **(B)**. Mean lifespan. **(C)**. Effects of CIEO or CID treatment on the transcription levels of dysregulated antimicrobial peptide related genes in *P. aeruginosa* PA14-infected nematodes with *pmk-1* RNAi. Exposure to *P. aeruginosa* commenced at the L4 stage and spanned a period of 24 h. Bars represent means ± SD. **p < 0.01 vs. control.

Molecular signals, including insulin and p38 MAPK have been identified as playing crucial roles in the regulation of bacterial infection (Zhi et al., 2017; Zhang et al., 2022). We subsequently investigated the potential involvement of these molecular signals in regulating the antibacterial effects of CID or CIEO in extending the lifespan of *P. aeruginosa* PA14-infected nematodes. Following *P. aeruginosa* PA14 infection, RNAi of *daf-16* did not alter the effect of CID or CIEO (10 mg/L) in increasing the lifespan of nematodes (Figures 5A,B). In contrast, RNAi of *pmk-1* notably suppressed the effect of CID or CIEO (10 mg/L) in extending the lifespan of nematodes after *P. aeruginosa* PA14 infection (Figures 5A,B). Thus, PMK-1, identified as a p38 MAPK in *C. elegans*, was essential for the antibacterial effects of CID or CIEO in prolonging lifespan following *P. aeruginosa* PA14 infection.

Furthermore, we also explored the potential involvement of PMK-1 in regulating the pharmacological effects of CID or CIEO on the dysregulated expression of antimicrobial peptide related genes in *P. aeruginosa* PA14-infected nematodes. Our results revealed that compared with wild-type nematodes, *pmk-1* RNAi substantially enhanced the expression of antimicrobial peptide genes, including *lys-1*and *lys-8*, in CIEO or CID-treated *P. aeruginosa* PA14-infected nematodes (Figure 5C). Thus, PMK-1 mediated p38 MAPK signaling is required for the antibacterial effects of CIEO or CID in *P. aeruginosa* PA14-infected nematodes.

### 3.6 Inhibitory effects of CIEO or CID on other common pathogenic bacteria *in vitro*

**FIGURE 6:**
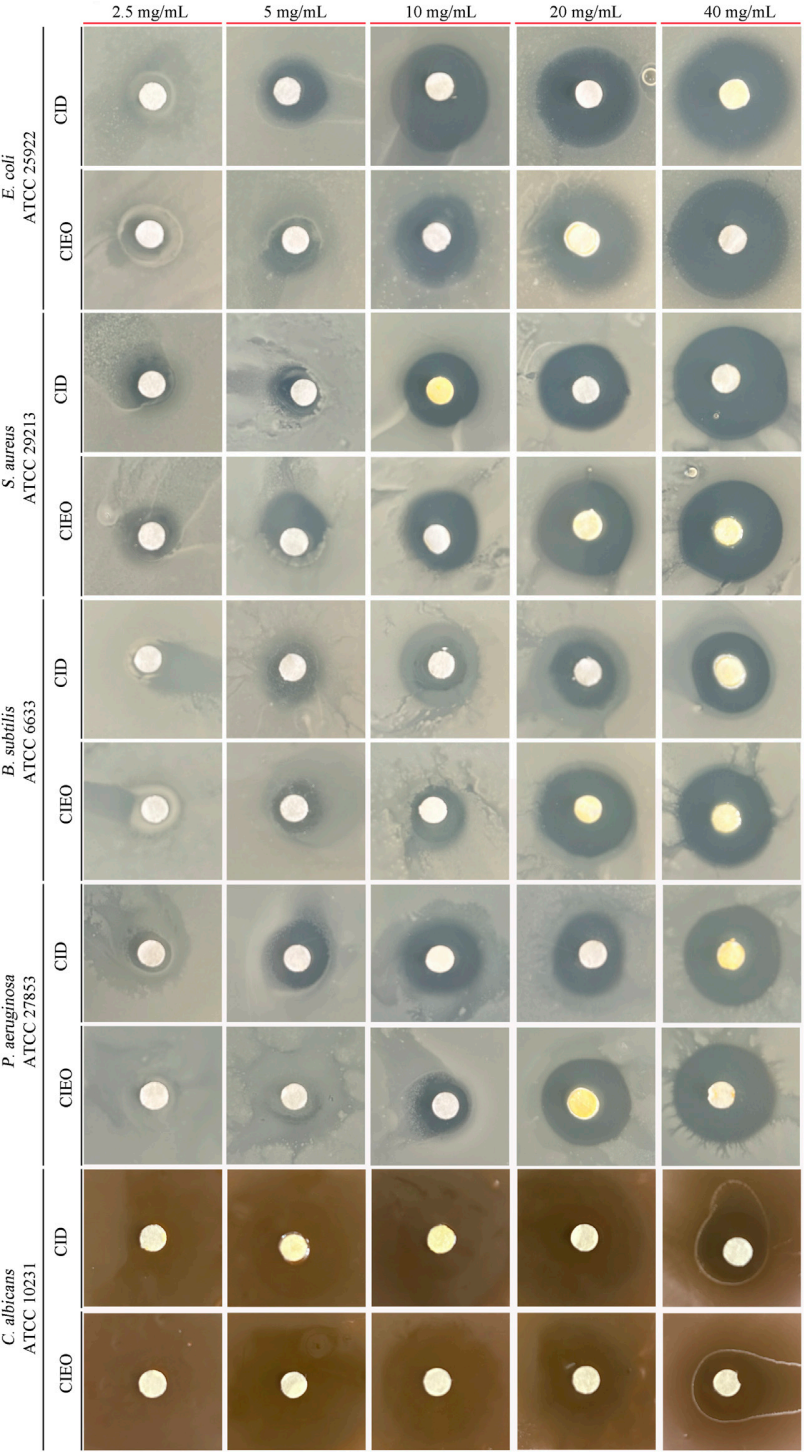
Determination of inhibition zone assay of CIEO and CID on *E. coli*, *S. aureus*, *B. subtilis*, *P. aeruginosa*, and *C. albicans*. The concentration of CIEO and CID ranged from 2.5 to 40 mg/mL.

To further investigate the *in vitro* antibacterial effects of CIEO and CID, we selected other common pathogenic bacteria, including *E. coli*, *S. aureus*, *B. subtilis*, and *C. albicans*, for *in vitro* antibacterial experiments. Our results revealed that CIEO and CID exhibited varying sensitivities against the five tested bacterial strains. As indicated in Figure 6 and Table 1, the antibacterial effects of CIEO and CID were positively correlated with their concentrations for the tested bacterial strains. The most effective inhibition is observed against *C. albicans*, followed by *S. aureus* and *E. coli*. At a concentration of 40 mg/mL, the inhibition zone diameters for CID against *C. albicans* and *S. aureus* were 37.90 ± 0.44 mm and 24.19 ± 0.11 mm, respectively, while those for CIEO are 36.77 ± 0.55 mm and 22.44 ± 0.13 mm. It could be inferred that the inhibitory effect of CID on *C. albicans* and *S. aureus* was superior to that of CIEO (Figure 6; Table 1).

**TABLE 1:** The inhibition zone (mm) of CIEO and CID.

Strain	Sample	Inhibition zone diameters (mm)				
		2.5 mg/mL	5 mg/mL	10 mg/mL	20 mg/mL	40 mg/mL
*E. coli* ATCC 25922	CID	9.42 ± 0.18	13.27 ± 0.42	19.58 ± 0.26	21.22 ± 0.09	24.37 ± 0.16
	CIEO	9.64 ± 0.15	9.81 ± 0.33	17.34 ± 0.12	22.75 ± 0.42	25.38 ± 0.18
*S. aureus* ATCC 29213	CID	9.44 ± 0.21	9.93 ± 0.20	16.58 ± 0.16	19.91 ± 0.40	24.19 ± 0.11
	CIEO	9.87 ± 0.30	13.27 ± 0.36	16.39 ± 0.11	20.27 ± 0.12	22.44 ± 0.13
*B. subtilis* ATCC 6633	CID	8.66 ± 0.39	10.51 ± 0.22	17.69 ± 0.16	14.43 ± 0.18	16.55 ± 0.21
	CIEO	7.29 ± 0.14	10.04 ± 0.41	12.52 ± 0.16	19.47 ± 0.25	20.59 ± 0.54
*P. aeruginosa* ATCC 27853	CID	9.19 ± 0.06	13.85 ± 0.23	17.28 ± 0.44	15.74 ± 0.44	20.44 ± 0.10
	CIEO	7.67 ± 0.44	9.27 ± 0.11	12.51 ± 0.16	19.34 ± 0.12	22.35 ± 0.09
*C. albicans* ATCC 10231	CID	9.27 ± 0.15	14.53 ± 0.67	17.50 ± 0.82	28.50 ± 0.10	37.90 ± 0.44
	CIEO	14.77 ± 0.31	15.90 ± 0.20	20.37 ± 0.21	23.50 ± 0.26	36.77 ± 0.55

According to Supplementary Tables S1, S3, the concentrations at which CIEO and CID inhibited the growth of *E. coli* were 0.62 mg/mL and 1.25 mg/mL, respectively. Conversely, for *S. aureus*, the inhibitory concentrations were 1.25 mg/mL and 0.62 mg/mL for CIEO and CID, respectively. Both CIEO and CID exhibited inhibitory concentrations of 0.31 mg/mL against *B. subtilis*. Concerning *P. aeruginosa*, the inhibitory concentrations stranded at 0.62 mg/mL and 0.31 mg/mL for CIEO and CID, respectively. Finally, against *C. albicans*, the inhibitory concentrations are 0.31 mg/mL for CIEO and 0.08 mg/mL for CID and, as shown in Table 2. The minimum bactericidal concentrations (MBC) of CIEO for *E. coli*, *S. aureus*, *B. subtilis*, *P. aeruginosa*, and *C. albicans* were 2.5, 10, 5, 5, and 0.62 mg/mL, respectively. Meanwhile, the MBC of CID for the same strains were 5, 5, 5, 2.5, and 0.31 mg/mL, respectively.

**TABLE 2:** MIC and MBC of CIEO and CID.

Sample	*E. coli* ATCC 25922		*S. aureus* ATCC 29213		*B. subtilis* ATCC 6633		*P. aeruginosa* ATCC 27853		*C. albicans* ATCC 10231	
	MIC mg/mL	MBC mg/mL	MIC mg/mL	MBC mg/mL	MIC mg/mL	MBC mg/mL	MIC mg/mL	MBC mg/mL	MIC mg/mL	MBC mg/mL
CID	1.25	5	0.62	5	0.31	5	0.31	2.5	0.08	0.31
CIEO	0.62	2.5	1.25	10	0.31	5	0.62	5	0.31	0.62

### 3.7 *In vitro* antioxidant capacity detection of CIEO or CID

**FIGURE 7:**
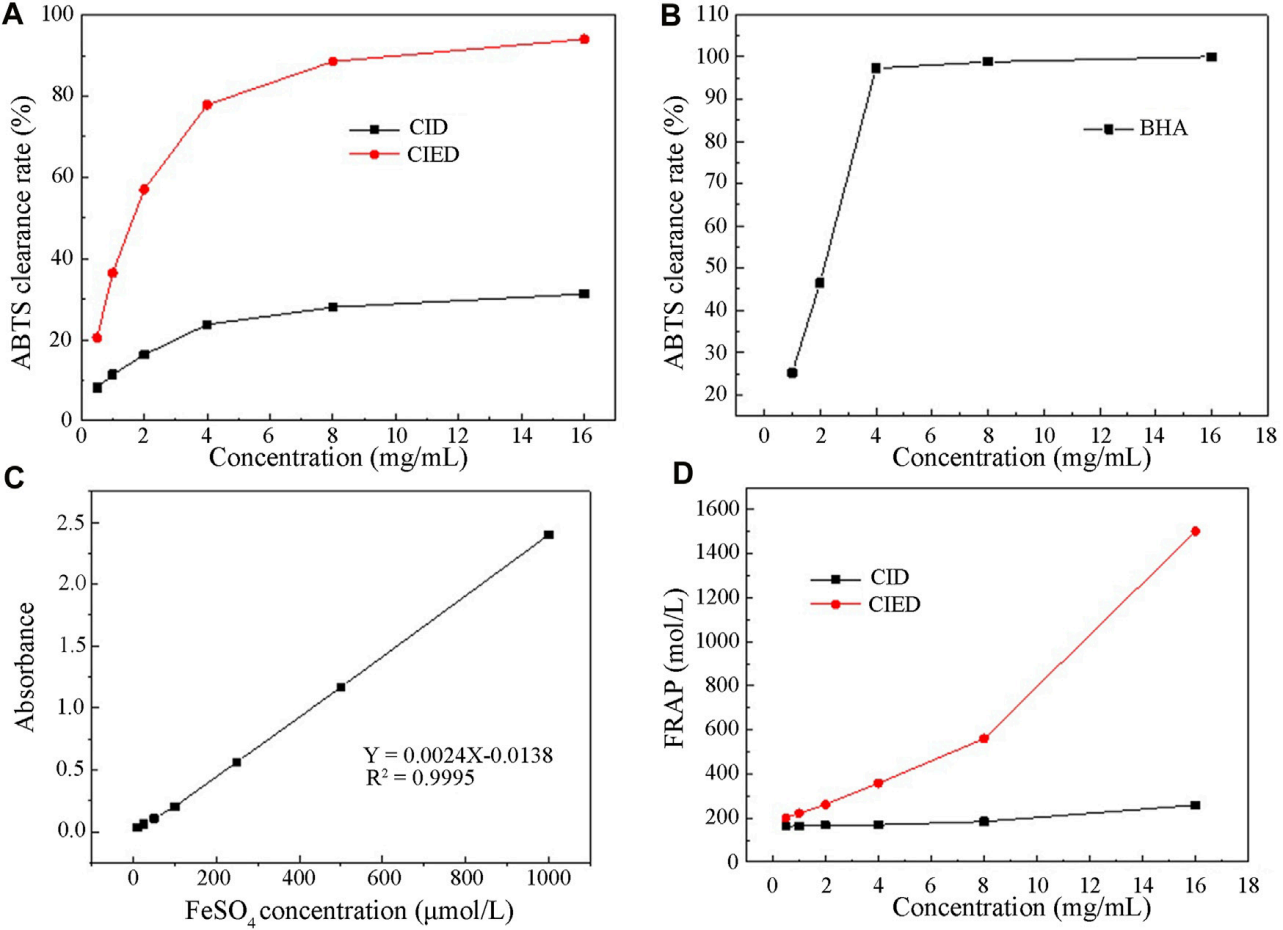
Antioxidant activity of CIEO and CID **(A)**. CIEO and CID clearance rate of ABTS free radicals **(B)**. BHA clearance rate of ABTS free radicals **(C)**. Standard curve of FeSO_4_ **(D)**. Fe^3+^ reduction ability of CIEO and CID.

Figure 7 depicted the change in clearance rates of ABTS free radicals with different concentrations of CIEO, CID, and BHA. Our results indicated that the clearance rate of CID remained relatively constant within the selected concentration range (0–18 mg/L), around 25% (Figure 7A). On the other hand, the clearance rate of CIEO increased with concentration, showing a positive correlation (Figure 7A). At a concentration of 16 mg/mL, the clearance capacity of CIEO reached 94.1%, 3 folds higher than the clearance rate of CID at the same concentration (Figure 7A). CIEO’s ability to clear ABTS was comparable to the effect of 4 mg/L BHA (Figures 7A,B).

In Figure 7C, the standard curve of FeSO_4_ illustrated a proportional relationship between FeSO_4_ concentration and absorbance within the range of 10–1,000 μmol/L. Figure 7D depicted the reducing power of CIEO and CID. Notably, the reducing power of CID remains relatively constant with minimal variation in concentration (Figure 7D). In contrast, the reducing power of CIEO exhibited an increase with concentration (Figure 7D). At a concentration of 16 mg/mL, the reducing power of CID corresponded to 258.6 μmol/L FeSO_4_, while the reducing power of CIEO at the same concentration equated to 1,502 μmol/L FeSO_4_, marking it as 5.8 folds higher than that of CID. This observation suggested that the antioxidant activity of CIEO surpassed that of CID.

## 4 Discussion

Infection with *P. aeruginosa* is linked to various infectious diseases, including sepsis, endocarditis, pneumonia, urinary tract infections, peritonitis, and other health hazards (Jacobs et al., 2020; Reyes et al., 2023). Recognized as one of the most formidable nosocomial pathogens, the treatment of *P. aeruginosa* infection poses a challenge due to the restricted array of available antibiotics (Bassetti et al., 2018). *C. elegans* has established itself as a robust invertebrate model for investigating host-pathogen interactions (Aballay and Ausubel, 2002; Yu et al., 2018; Martineau et al., 2021; Wang et al., 2023). CIEO and its main component, CID, have been confirmed to possess various biological activities, including antiplatelet activity, anti-hyperglycemic and lipid-lowering applications, inhibition of cancer cell proliferation, and antibacterial properties (Ka et al., 2003; Tognolini et al., 2006; Subash Babu et al., 2007; Lu et al., 2020; Abeysekera et al., 2022). However, the *in vivo* and *in vitro* antibacterial mechanisms of CIEO and CID remain unclear.

In the present investigation, we employed the *in vivo* evaluation model utilizing *C. elegans*, with locomotion behavior and lifespan serving as parameters to assess the *in vivo* antibacterial effects of CIEO and CID. Our results revealed that wild-type nematodes, upon infection with *P. aeruginosa* PA14, underwent a notable decrease in both locomotion ability and lifespan (Figure 2). Treatment with CIEO and CID in *P. aeruginosa* PA14-infected *C. elegans* effectively mitigated the decline in locomotion ability and lifespan induced by the infection, and this reversal process demonstrated a dose-dependent effect (Figure 2). Furthermore, utilizing the accumulation of CFU and PA::GFP in nematodes as evaluation indicators, we observed a significant increase in CFU and PA::GFP accumulation in wild-type nematodes infected with *P. aeruginosa* PA14 compared to the control group (Figure 3). Treatment with CIEO and CID in *P. aeruginosa* PA14-infected nematodes successfully inhibited the rise in CFU and PA::GFP accumulation caused by the *P. aeruginosa* PA14 infection, with this inhibitory effect positively correlating with the dosage of CIEO and CID (Figure 3).

We then investigated the *in vivo* antibacterial mechanisms of CIEO and CID on *C. elegans* infected with *P. aeruginosa* PA14. Interestingly, we observed distinct antibacterial mechanisms between the low dose (10 mg/L) and high dose (100 mg/L) groups of CIEO and CID. Treatment with low doses (10 mg/L) of CIEO and CID in *P. aeruginosa* PA14-infected wild-type nematodes significantly upregulated the expression of antimicrobial peptide genes *lys-1* and *lys-8* (Figure 4). In contrast, the expression levels of these genes or proteins did not show further elevation with high doses (100 mg/L) of CIEO and CID treatment in *P. aeruginosa* PA14-infected wild-type *C. elegans* (Figure 4). Therefore, we postulate that the antibacterial mechanisms of low doses (10 mg/L) of CIEO and CID in *P. aeruginosa* PA14-infected wild-type *C. elegans* may involve the activation of the host’s innate immune. In contrast, the lack of further activation of these systems in the high dose (100 mg/L) groups of CIEO and CID may be attributed to their direct inhibitory effects on *P. aeruginosa* PA14. We further investigated the impact of *daf-16* and *pmk-1* RNAi on the beneficial effects of 10 mg/L CIEO or CID treatment in *P. aeruginosa* PA14-infected nematodes. RNAi of *pmk-1* notably attenuated the effectiveness of CID or CIEO (10 mg/L) in extending the lifespan of nematodes after *P. aeruginosa* PA14 infection (Figure 5). Additionally, *pmk-1* RNAi significantly increased the expression of antimicrobial peptide genes, including *lys-1* and *lys-8*, in CIEO or CID-treated *P. aeruginosa* PA14-infected nematodes (Figure 5). Thus, PMK-1, recognized as a p38 MAPK in *C. elegans*, played a crucial role in the antibacterial effects of CID or CIEO, contributing to the extension of lifespan following *P. aeruginosa* PA14 infection.

Subsequent investigations were carried out to assess the antibacterial activity of CIEO and CID utilizing the filter paper disc method, MIC, and MBC. The findings revealed that both substances displayed more pronounced inhibitory effects on fungi as opposed to bacteria (Table 2). Specifically, CID exhibited superior inhibitory effects against *S. aureus*, *C. albicans*, and *P. aeruginosa* when compared to CIEO (Table 2). Conversely, CIEO demonstrated enhanced antibacterial effects against *E. coli* in comparison to CID (Table 2). A recent study showed that β-caryophyllene could enhance antioxidant activity, indicating a synergistic effect among the antioxidant components in CIEO containing β-caryophyllene (Yovas et al., 2023), which explained why the antibacterial effect of CIEO is stronger than that of CID against certain specific pathogens. The antioxidant activities of CIEO and CID were compared using the ABTS radical scavenging assay and FRAP determination. Our study revealed significant differences in the antioxidant activities of the two substances. At a concentration of 16 mg/mL, the ABTS scavenging ability of CIEO was 94.1%, 3 folds higher than that of CID at the same concentration (Figure 7). The FRAP value of CIEO was 1,502 μmol/L, 5.8 folds higher than that of CID at the equivalent concentration (Figure 7). Several research highlights a robust positive correlation between the antioxidant activity of a substance and its antibacterial properties (Martelli and Giacomini, 2018; Li et al., 2022; Lorenzo et al., 2022). The underlying mechanism suggests that the substance exerts antibacterial effects through its anti-free radical activity (Lorenzo et al., 2022). Our results indicated that CIEO and CID displayed potent antioxidant activity (Figure 7). As such, we posit that this attribute may contribute to the broad-spectrum antibacterial effects observed in CIEO and CID.

Existing studies suggest that CIEO and CID possesses broad-spectrum antibacterial activity against both Gram-positive and Gram-negative bacteria (Cheng et al., 2012). At a concentration of 500 ppm, CIEO can inhibit the growth of various bacterial strains, including *Streptococcus*, *Enterococcus*, *Bacillus*, *Clostridium*, *Listeria*, and *Candida* species (Miyashita and Sadzuka, 2013). CIEO (0.05%) effectively inhibits the growth of *E. coli*, and the inhibitory effect is dependent on the initial quantity of bacterial strains and populations (Sheng and Zhu, 2014). The MIC of CIEO ranges from 200 to 400 mg/L, with an MBC ranging from 400 to 800 mg/L (Becerril et al., 2012). CIEO is considered an ideal antibacterial substance as it does not induce the development of resistance and remains effective even after prolonged use (Pina-Pérez et al., 2012). In white wine, adding 2 g of cinnamon is more effective in inhibiting *H. pylori* than green tea and raspberries (Lin et al., 2005). Cinnamon can also inhibit the growth of *Listeria* monocytogenes in meat products, with inhibitory effects superior to those of garlic and thyme (Dussault et al., 2014). In a study by Manso et al., 2013, volatile compounds in CIEO induced hyphal disruption in *Aspergillus flavus*, forming irregular cell clusters that seemed to be a form of heterogeneous aggregate, significantly impacting spore inhibition. In their research, CIEO led to incomplete formation of conidia, inhibiting growth. Manso et al., 2013 suggests that the impact of the main component of CIEO, CID, on enzyme activity enhances its anti-toxigenic activity against *A. flavus*. By blocking the enzymatic activity of biosynthetic enzymes, it can inhibit the formation of aflatoxins (fungal toxins produced by *A. flavus*, increasing the risk of liver cell cancer). Alpsoy, 2010 reported that CIEO can reduce the pathogenic effects of aflatoxin by either decreasing the binding of aflatoxin to DNA or reacting with the reactive oxygen species (ROS) produced by aflatoxin.

Although some progress has been made in understanding the antibacterial effects and potential mechanisms of CIEO and its main component, CID, further research is still needed to fully comprehend the antibacterial effects and mechanisms within living organisms. Our study employed the nematode *C. elegans* as an *in vivo* model to systematically analyze the antibacterial mechanisms of CIEO and CID. The combination of *in vitro* antibacterial effect evaluations and exploration of potential mechanisms provides scientific evidence for the use of CIEO and CID as antibacterial agents.

## 5 Conclusion

Taken together, in this study, we utilized *C. elegans* as a host model to investigate the *in vivo* potential antibacterial effects and mechanisms of CIEO and CID treatment against *P. aeruginosa* infection. We also determined the *in vitro* antibacterial effects of CIEO and CID, along with their potential mechanisms. We found that both high and low doses of CIEO and CID treatment significantly alleviated the reduction in motility, lifespan, and accumulation of *P. aeruginosa* in *C. elegans* infected with the bacteria. However, the mechanisms of action differed between high and low-dose treatments. Based on our results, we proposed that low-dose treatment during *P. aeruginosa* infection in *C. elegans* could promote the transcriptional expression of antimicrobial peptides. Further investigation suggested that the PMK-1 mediated p38 signaling pathway may play a role in regulating CIEO and CID during nematode defense against *P. aeruginosa* infection. Additionally, *in vitro* antibacterial experiments and antioxidant capacity measurements further confirmed that CIEO and CID could serve as potential antibacterial agents. Our data suggest the potential of CIEO and CID in inhibiting pathogenic infections both *in vivo* and *in vitro*.

## Data Availability

The raw data supporting the conclusion of this article will be made available by the authors, without undue reservation.

## References

[B1] Aballay A. Ausubel F. M. (2002). *Caenorhabditis elegans* as a host for the study of host-pathogen interactions. Curr. Opin. Microbiol. 5, 97–101. 10.1016/s1369-5274(02)00293-x 11834377

[B2] Abeysekera WPKM. Premakumara G. A. S. Ratnasooriya W. D. Abeysekera WKSM. (2022). Anti-inflammatory, cytotoxicity and antilipidemic properties: novel bioactivities of true cinnamon (Cinnamomum zeylanicum Blume) leaf. BMC Complement. Med. Ther. 22, 259. 10.1186/s12906-022-03728-5 36195907 PMC9531470

[B3] Alper S. McBride S. J. Lackford B. Freedman J. H. Schwartz D. A. (2007). Specificity and complexity of the *Caenorhabditis elegans* innate immune response. Mol. Cell. Biol. 27, 5544–5553. 10.1128/MCB.02070-06 17526726 PMC1952075

[B4] Alpsoy L. (2010). Inhibitory effect of essential oil on aflatoxin activities. Afr. J. Biotechnol. 9, 2474–2481.

[B5] Andrews J. M. (2001). Determination of minimum inhibitory concentrations. J. Antimicrob. Chemother. 1, 5–16. 10.1093/jac/48.suppl_1.5 11420333

[B6] Bassetti M. Vena A. Russo A. Croxatto A. Calandra T. Guery B. (2018). Rational approach in the management of *Pseudomonas aeruginosa* infections. Curr. Opin. Infect. Dis. 31, 578–586. 10.1097/QCO.0000000000000505 30299364

[B7] Becerril R. Nerín C. Gómez-Lus R. (2012). Evaluation of bacterial resistance to essential oils and antibiotics after exposure to oregano and cinnamon essential oils. Foodborne Pathog. Dis. 9, 699–705. 10.1089/fpd.2011.1097 22827568

[B8] Chelliah R. Choi J. G. Hwang S. B. Park B. J. Daliri E. B. Kim S. H. (2018). *In vitro* and *in vivo* defensive effect of probiotic LAB against *Pseudomonas aeruginosa* using *Caenorhabditis elegans* model. Virulence 9, 1489–1507. 10.1080/21505594.2018.1518088 30257614 PMC6177248

[B9] Chen L. Sun P. Wang T. Chen K. Jia Q. Wang H. (2012). Diverse mechanisms of antidiabetic effects of the different procyanidin oligomer types of two different cinnamon species on db/db mice. J. Agric. Food Chem. 60, 9144–9150. 10.1021/jf3024535 22920511

[B10] Cheng D. M. Kuhn P. Poulev A. Rojo L. E. Lila M. A. Raskin I. (2012). *In vivo* and *in vitro* antidiabetic effects of aqueous cinnamon extract and cinnamon polyphenol-enhanced food matrix. Food Chem. 135, 2994–3002. 10.1016/j.foodchem.2012.06.117 22980902 PMC3444749

[B11] Du H. Bing J. Hu T. Ennis C. L. Nobile C. J. Huang G. (2020). Candida auris: epidemiology, biology, antifungal resistance, and virulence. PLoS Pathog. 16, e1008921. 10.1371/journal.ppat.1008921 33091071 PMC7581363

[B12] Dussault D. Vu K. D. Lacroix M. (2014). *In vitro* evaluation of antimicrobial activities of various commercial essential oils, oleoresin and pure compounds against food pathogens and application in ham. Meat Sci. 96, 514–520. 10.1016/j.meatsci.2013.08.015 24012976

[B13] Fernandes C. J. Stevens D. A. Groot obbink D. J. Ackerman V. P. (1985). A replicator method for the combined determination of minimum inhibitory concentration and minimum bactericidal concentration. J. Antimicrob. Chemother. 15, 53–60. 10.1093/jac/15.1.53 3972758

[B14] Floridi A. Piroddi M. Pilolli F. Matsumoto Y. Aritomi M. Galli F. (2009). Analysis method and characterization of the antioxidant capacity of vitamin E-interactive polysulfone hemodialyzers. Acta Biomater. 5, 2974–2982. 10.1016/j.actbio.2009.04.011 19442768

[B15] Gruenwald J. Freder J. Armbruester N. (2010). Cinnamon and health. Crit. Rev. Food Sci. Nutr. 50, 822–834. 10.1080/10408390902773052 20924865

[B16] Jacobs D. M. Ochs-Balcom H. M. Noyes K. Zhao J. Leung W. Y. Pu C. Y. (2020). Impact of *Pseudomonas aeruginosa* isolation on mortality and outcomes in an outpatient chronic obstructive pulmonary disease cohort. Open Forum Infect. Dis. 7, ofz546. 10.1093/ofid/ofz546 31993457 PMC6979313

[B17] Ka H. Park H. J. Jung H. J. Choi J. W. Cho K. S. Ha J. (2003). Cinnamaldehyde induces apoptosis by ROS-mediated mitochondrial permeability transition in human promyelocytic leukemia HL-60 cells. Cancer Lett. 196, 143–152. 10.1016/s0304-3835(03)00238-6 12860272

[B18] Kamath R. S. Ahringer J. (2003). Genome-wide RNAi screening in *Caenorhabditis elegans* . Methods 30, 313–321. 10.1016/s1046-2023(03)00050-1 12828945

[B19] Li A. Li L. Zhao B. Li X. Liang W. Lang M. (2022). Antibacterial, antioxidant and anti-inflammatory PLCL/gelatin nanofiber membranes to promote wound healing. Int. J. Biol. Macromol. 194, 914–923. 10.1016/j.ijbiomac.2021.11.146 34838860

[B20] Lin Y. T. Vattem D. Labbe R. G. Shetty K. (2005). Enhancement of antioxidant activity and inhibition of *Helicobacter pylori* by phenolic phytochemical-enriched alcoholic beverages. Process Biochem. 40, 2059–2065. 10.1016/j.procbio.2004.07.019

[B21] Lorenzo M. E. Casero C. N. Gómez P. E. Segovia A. F. Figueroa L. C. Quiroga A. (2022). Antioxidant characteristics and antibacterial activity of native woody species from Catamarca, Argentina. Nat. Prod. Res. 36, 885–890. 10.1080/14786419.2020.1839461 33185143

[B22] Lu L. Shu C. Chen L. Yang Y. Ma S. Zhu K. (2020). Insecticidal activity and mechanism of cinnamaldehyde in *C. elegans* . Fitoterapia 146, 104687. 10.1016/j.fitote.2020.104687 32681860

[B23] Ma C. Xue T. Peng Q. Zhang J. Guan J. Ding W. (2023). A novel N6-Deoxyadenine methyltransferase METL-9 modulates *C. elegans* immunity via dichotomous mechanisms. Cell Res. 33, 628–639. 10.1038/s41422-023-00826-y 37271765 PMC10397248

[B24] Mallo G. V. Kurz C. L. Couillault C. Pujol N. Granjeaud S. Kohara Y. (2002). Inducible antibacterial defense system in *C. elegans* . Curr. Biol. 12, 1209–1214. 10.1016/s0960-9822(02)00928-4 12176330

[B25] Manso S. Cacho-Nerin F. Becerril R. Nerín C. (2013). Combined analytical and microbiological tools to study the effect on Aspergillus flavus of cinnamon essential oil contained in food packaging. Food Control 30, 370–378. 10.1016/j.foodcont.2012.07.018

[B26] Martelli G. Giacomini D. (2018). Antibacterial and antioxidant activities for natural and synthetic dual-active compounds. Eur. J. Med. Chem. 158, 91–105. 10.1016/j.ejmech.2018.09.009 30205261

[B27] Martineau C. N. Kirienko N. V. Pujol N. (2021). Innate immunity in *C. elegans* . Curr. Top. Dev. Biol. 144, 309–351. 10.1016/bs.ctdb.2020.12.007 33992157 PMC9175240

[B28] Miyashita M. Sadzuka Y. (2013). Effect of linalool as a component of Humulus lupulus on doxorubicin-induced antitumor activity. Food Chem. Toxicol. 53, 174–179. 10.1016/j.fct.2012.11.035 23220514

[B29] Pina-Pérez M. C. Martínez-López A. Rodrigo D. (2012). Cinnamon antimicrobial effect against *Salmonella typhimurium* cells treated by pulsed electric fields (PEF) in pasteurized skim milk beverage. Food Res. Int. 48, 777–783. 10.1016/j.foodres.2012.06.027

[B30] Qu M. An Y. Jiang X. Wu Q. Miao L. Zhang X. (2023c). Exposure to epoxy-modified nanoplastics in the range of μg/L causes dysregulated intestinal permeability, reproductive capacity, and mitochondrial homeostasis by affecting antioxidant system in *Caenorhabditis elegans* . Aquat. Toxicol. 264, 106710. 10.1016/j.aquatox.2023.106710 37804785

[B31] Qu M. Chen H. Lai H. Liu X. Wang D. Zhang X. (2022). Exposure to nanopolystyrene and its 4 chemically modified derivatives at predicted environmental concentrations causes differently regulatory mechanisms in nematode *Caenorhabditis elegans* . Chemosphere 305, 135498. 10.1016/j.chemosphere.2022.135498 35777546

[B32] Qu M. Miao L. Chen H. Zhang X. Wang Y. (2023b). SKN-1/Nrf2-dependent regulation of mitochondrial homeostasis modulates transgenerational toxicity induced by nanoplastics with different surface charges in *Caenorhabditis elegans* . J. Hazard. Mat. 457, 131840. 10.1016/j.jhazmat.2023.131840 37327611

[B33] Qu M. Miao L. Liu X. Lai H. Hao D. Zhang X. (2023a). Organismal response to micro(nano)plastics at environmentally relevant concentrations: toxicity and the underlying mechanisms. Ecotoxicol. Environ. Saf. 254, 114745. 10.1016/j.ecoenv.2023.114745 36950991

[B34] Qu M. Qiu Y. Lv R. Yue Y. Liu R. Yang F. (2019). Exposure to MPA-capped CdTe quantum dots causes reproductive toxicity effects by affecting oogenesis in nematode *Caenorhabditis elegans* . Ecotoxicol. Environ. Saf. 173, 54–62. 10.1016/j.ecoenv.2019.02.018 30769203

[B35] Qu M. Xu K. Li Y. Wong G. Wang D. (2018). Using acs-22 mutant *Caenorhabditis elegans* to detect the toxicity of nanopolystyrene particles. Sci. Total Environ. 643, 119–126. 10.1016/j.scitotenv.2018.06.173 29936155

[B36] Qu M. Zhao X. Wang Q. Xu X. Chen H. Wang Y. (2024). PIEZO mediates a protective mechanism for nematode *Caenorhabditis elegans* in response to nanoplastics caused dopaminergic neurotoxicity at environmentally relevant concentrations. Ecotoxicol. Environ. Saf. 269, 115738. 10.1016/j.ecoenv.2023.115738 38056120

[B37] Reyes L. F. Garcia E. Ibáñez-Prada E. D. Serrano-Mayorga C. C. Fuentes Y. V. Rodríguez A. (2023). Impact of macrolide treatment on long-term mortality in patients admitted to the ICU due to CAP: a targeted maximum likelihood estimation and survival analysis. Crit. Care. 27, 212. 10.1186/s13054-023-04466-x 37259125 PMC10230128

[B38] Sathe N. Beech P. Croft L. Suphioglu C. Kapat A. Athan E. (2023). *Pseudomonas aeruginosa*: infections and novel approaches to treatment "Knowing the enemy" the threat of *Pseudomonas aeruginosa* and exploring novel approaches to treatment. Infect. Med. 2, 178–194. 10.1016/j.imj.2023.05.003 PMC10699684 38073886

[B39] Shao H. Wang D. (2020). Long-term and low-dose exposure to nanopolystyrene induces a protective strategy to maintain functional state of intestine barrier in nematode *Caenorhabditis elegans* . Environ. Pollut. 258, 113649. 10.1016/j.envpol.2019.113649 31767235

[B40] Shapira M. Hamlin B. J. Rong J. Chen K. Ronen M. Tan M. W. (2006). A conserved role for a GATA transcription factor in regulating epithelial innate immune responses. Proc. Natl. Acad. Sci. 103, 14086–14091. 10.1073/pnas.0603424103 16968778 PMC1599916

[B41] Sheng L. Zhu M. (2014). Inhibitory effect of Cinnamomum cassia oil on non-O157 Shiga toxin-producing *Escherichia coli* . Food Control 46, 374–381. 10.1016/j.foodcont.2014.05.050

[B42] Sim J. X. F. Khazandi M. Pi H. Venter H. Trott D. J. Deo P. (2019). Antimicrobial effects of cinnamon essential oil and cinnamaldehyde combined with EDTA against canine otitis externa pathogens. J. Appl. Microbiol. 127, 99–108. 10.1111/jam.14298 31050849

[B43] Stover C. K. Pham X. Q. Erwin A. L. Mizoguchi S. D. Warrener P. Hickey M. J. (2000). Complete genome sequence of *Pseudomonas aeruginosa* PAO1, an opportunistic pathogen. Nature 406, 959–964. 10.1038/35023079 10984043

[B44] Subash Babu P. Prabuseenivasan S. Ignacimuthu S. (2007). Cinnamaldehyde--a potential antidiabetic agent. Phytomedicine 14, 15–22. 10.1016/j.phymed.2006.11.005 17140783

[B45] Tognolini M. Barocelli E. Ballabeni V. Bruni R. Bianchi A. Chiavarini M. (2006). Comparative screening of plant essential oils: phenylpropanoid moiety as basic core for antiplatelet activity. Life Sci. 78, 1419–1432. 10.1016/j.lfs.2005.07.020 16274702

[B46] Wang Y. Zhang L. Yuan X. Wang D. (2023). Treatment with paeoniflorin increases lifespan of *Pseudomonas aeruginosa* infected *Caenorhabditis elegans* by inhibiting bacterial accumulation in intestinal lumen and biofilm formation. Front. Pharmacol. 14, 1114219. 10.3389/fphar.2023.1114219 37050896 PMC10083309

[B47] White C. V. Herman M. A. (2018). Transcriptomic, functional, and network analyses reveal novel genes involved in the interaction between *Caenorhabditis elegans* and stenotrophomonas maltophilia. Front. Cell. Infect. Microbiol. 8, 266. 10.3389/fcimb.2018.00266 30177956 PMC6109753

[B48] Yang W. H. Chen P. H. Chang H. H. Kwok H. L. Stern A. Soo P. C. (2023). Impaired immune response and barrier function in GSPD-1-deficient *C. elegans* infected with *Klebsiella pneumoniae* . Curr. Res. Microb. Sci. 4, 100181. 10.1016/j.crmicr.2023.100181 36798906 PMC9926097

[B49] Yovas A. Stanely S. P. Ponnian S. M. P. (2023). Protective effects of β-caryophyllene on mitochondrial damage and cardiac hypertrophy pathways in isoproterenol-induced myocardial infarcted rats. Eur. J. Pharmacol. 952, 175785. 10.1016/j.ejphar.2023.175785 37207967

[B50] Yu Y. Zhi L. Wu Q. Jing L. Wang D. (2018). NPR-9 regulates the innate immune response in *Caenorhabditis elegans* by antagonizing the activity of AIB interneurons. Cell. Mol. Immunol. 15, 27–37. 10.1038/cmi.2016.8 27133473 PMC5827173

[B51] Zhang L. Wang Y. Cao C. Zhu Y. Huang W. Yang Y. (2022). Beneficial effect of Xuebijing against *Pseudomonas aeruginosa* infection in *Caenorhabditis elegans* . Front. Pharmacol. 13, 949608. 10.3389/fphar.2022.949608 36120363 PMC9470999

[B52] Zhi L. Yu Y. Jiang Z. Wang D. (2017). mir-355 functions as an important link between p38 MAPK signaling and insulin signaling in the regulation of innate immunity. Sci. Rep. 7, 14560. 10.1038/s41598-017-15271-2 29109437 PMC5673931

