# Supplementary material for: Antibacterial activity of cinnamon essential oil and its main component of cinnamaldehyde and the underlying mechanism
Source: Front Pharmacol. 2024 Mar 11;15:1378434. doi: 10.3389/fphar.2024.1378434 (PMC10961361; doi:10.3389/fphar.2024.1378434)
Supplement: Supplementary file 1 [file Table1.doc]

**Supporting Information:**

**Table. S1.** Primer information for qRT-PCR

| Gene | Forward primer (5’-3’) | Reverse primer (5’-3’) |
| --- | --- | --- |
| *lys-1* | TTCGGATCTTTCAAGAAG | TGGGATTCCAACAACGTA |
| *lys-8* | TCAGTCTCCGTCAAGGTC | GAAGCTGGCTCAATGAAA |
| *clec-85* | GGTTTTGGCTGTAGCACG | GGTTTTGGCTGTAGCACG |
| *dod-22* | CCAGGATACAGAATACGT | CCAGAGATGACTTCAGTT |
| *K08D8.5* | TTACGATGGTGATTCCGT | GCTTGTTGCCAGTTGAGA |
| *F55G11.7* | CACCCTCAGGCCAACTCA | CTGTGACTGTAGCGTCAC |
| *F55G11.4* | GGATCCGTGTATTTGGCT | GTGAAGACATATGTGCTC |
| *tba-1* | TCAACACTGCCATCGCCGCC | TCCAAGCGAGACCAGGCTTCAG |

**Table. S2.** Primer information for RNAi

| Gene | Forward primer (5’-3’) | Reverse primer (5’-3’) |
| --- | --- | --- |
| *daf-16* | AGTACAGCAATTCCCAAATGAAA | AATTGGATTTCGAAGAAGTGGAT |
| *pmk-1* | CAGGTAACGGAGCCAATGTT | GCGCCTAAATCCTCAAATCA |

**Table. S3 GC-MS analysis of CIEO**

| **Peak No.** | **Compounds** | ***t*_R_ (min)** | **Relative content**  **(%)** | **Matching**  **(%)** |
| --- | --- | --- | --- | --- |
| 1 | Styrene | 4.620 | 0.42 | 95 |
| 2 | (1S)-(-)-α-Pinene | 5.484 | 0.19 | 97 |
| 3 | Camphor | 5.873 | 0.14 | 97 |
| 4 | Benzaldehyde | 6.297 | 0.48 | 91 |
| 5 | Eucalyptol | 8.134 | 0.40 | 96 |
| 6 | Borneol | 12.494 | 0.12 | 94 |
| 7 | Cinnamaldehyde | 14.153 | 86.07 | 97 |
| 8 | α-Copaene | 18.502 | 1.04 | 99 |
| 9 | Caryophyllene | 19.738 | 0.22 | 99 |
| 10 | Coumarin | 20.722 | 0.67 | 94 |
| 11 | γ-Muurolene | 21.208 | 0.41 | 99 |
| 12 | 2-Methoxycinnamaldehyde | 22.925 | 6.25 | 98 |
| 13 | Diisobutyl phthalate | 31.599 | 1.02 | 90 |
| 14 | Cyclohexadecane | 33.694 | 0.46 | 98 |

| **Strain** | **Sample** | **Concentration mg/mL** | | | | | | | | | **Control** |
| --- | --- | --- | --- | --- | --- | --- | --- | --- | --- | --- | --- |
|  |  | **10** | **5** | **2.5** | **1.25** | **0.62** | **0.31** | **0.16** | **0.08** | **0.04** |  |
| *E. coli*  ATCC 25922 | CID | - | - | - | - | + | + | + | + | + | + |
|  | CIEO | - | - | - | - | - | + | + | + | + | + |
| *S. aureus*  ATCC 29213 | CID | - | - | - | - | - | + | + | + | + | + |
|  | CIEO | - | - | - | - | + | + | + | + | + | + |
| *B. subtilis*  ATCC  6633 | CID | - | - | - | - | - | - | + | + | + | + |
|  | CIEO | - | - | - | - | - | - | + | + | + | + |
| *P. aeruginosa*  ATCC 27853 | CID | - | - | - | - | - | - | + | + | + | + |
|  | CIEO | - | - | - | - | - | + | + | + | + | + |
| *C. albicans* ATCC 10231 | CID | - | - | - | - | - | - | - | - | + | + |
|  | CIEO | - | - | - | - | - | - | + | + | + | + |

**Table S4**. Minimum inhibitory concentration results
